# Impact of anodal tDCS and virtual reality on cognitive dysfunction in patients with Multiple Sclerosis: Protocol of a double blind, randomized, prospective, controlled study

**DOI:** 10.1371/journal.pone.0337405

**Published:** 2025-12-04

**Authors:** Lucilla Vestito, Cristina Schenone, Francesca Casazza, Roberto Modenesi, Angelo Iandolino, Maria Giuseppina Matichecchia, Lisbet Montes Saez, Massimiliano Botto, Marta Ponzano, Paola Gazzola, Erica Grange, Giampaolo Brichetto, Fabio Bandini, Carlo Trompetto, Laura Mori

**Affiliations:** 1 IRCCS Ospedale Policlinico San Martino, Genoa, Italy; 2 Department of Neuroscience, Rehabilitation, Ophthalmology, Genetics, Maternal and Child Health (DINOGMI), University of Genoa, Italy; 3 Department of Health Sciences (DISSAL), University of Genoa, Italy; 4 Scientific Research Area, Italian Multiple Sclerosis Foundation (FISM),; 5 AISM Rehabilitation Service, Italian Multiple Sclerosis Society, Genoa, Italy; 6 Department of Neurology, ASL 3 Genovese, Genoa, Italy; PLoS ONE, UNITED STATES OF AMERICA

## Abstract

**Background:**

Cognitive impairment (CI) affects a significant proportion of people with Multiple Sclerosis (PwMS), even in the early stages of the disease, increasing fatigue perception and reducing quality of life. Various restorative interventions have been tested to mitigate CI, but their efficacy remains limited. Transcranial direct current stimulation (tDCS) has emerged as a promising add-on to conventional rehabilitation in several neurological conditions, including MS. Similarly, novel technologies such as exergames are gaining attention as supportive tools in the rehabilitation of PwMS. These approaches can be combined within integrated protocols to maximize therapeutic benefits on CI. However, robust evidence of efficacy is still lacking, and only one case report has been published to date.

**Aims:**

The primary aim is to investigate the potential benefits of combining anodal tDCS (A-tDCS) with exergame-based training in the rehabilitation of CI in PwMS. The secondary aim is to assess whether the proposed protocol can provide sustained benefits over time.

**Methods:**

80 PwMS with CI will be consecutively enrolled and evaluated through a comprehensive neuropsychological battery. 40 participants will be randomized to the experimental group (EG) and 40 to the control group (CG). All participants will undergo 10 sessions of exergame-based rehabilitation. In addition, the EG will receive concurrent A-tDCS over the left dorsolateral prefrontal cortex, whereas the CG will receive sham stimulation. Assessments will be conducted at baseline, post-treatment, and at one and six months follow-up.

**Discussion:**

We hypothesize cognitive improvements in both groups due to exergame training. However, we expect greater improvements in the EG compared with the CG. Furthermore, we hypothesize that the beneficial effects in the EG will be sustained over time.

**Clinical trials registration:**

ClinicalTrials.gov, Identifier: NCT07114809 (Date: August 8th, 2025)

## Introduction

Cognitive impairment (CI) is one of the many symptoms that can affect individuals with Multiple Sclerosis (MS), potentially compromising activities of daily living (ADLs) and social functioning. Many papers investigate this aspect of the disease, documenting that cognitive dysfunction occurs in a high percentage of cases, ranging from 40% to 82%. The possible therapeutic approaches are mostly neuropsychological rehabilitation treatment, since pharmacological and immunosuppressive therapies did not show significant results [1]. Rehabilitation aims to increase the patient’s awareness of their own CI and create mechanisms for them to manage with CI in daily life, but especially to reduce cognitive deficits through cognitive retraining. Before undergoing the rehabilitation treatment, it is very important to accurately test the cognitive sphere to clearly identify if the impairment is in attention, working memory, information processing speed, visuospatial or executive deficits [2]. In recent years, transcranial direct current stimulation (tDCS) has emerged as a non-conventional tool in restorative neurology due to its ability in enhancing the efficacy of conventional therapeutic interventions. TDCS treatment consists of applying a direct current flow of low intensity (1–2 mA) over the scalp to modulate cortical excitability, by facilitating or inhibiting ongoing neuronal processes. Particularly, the anodal stimulation (A-tDCS) promotes mechanisms that underlie long-term potentiation and triggers widespread cortical activation [3]. The procedure is well-tolerated, with no evidence of risk for serious adverse effects and no associated risk of seizures [4]. TDCS has been reported to exert beneficial effects on pain, fatigue, tactile perception, cognition, attention and executive function [4–9]. Mattioli and coworkers [8] observed a beneficial effect of A-tDCS over the DLPFC on some cognitive domains. Grigorescu [10], on the other hand, found that bifrontal tDCS over the prefrontal cortex (PFC) did not lead to any effect on cognitive measures. These somehow conflicting studies show that further research is needed to better understand the potential role of tDCS in improving the cognitive performance in people with multiple sclerosis (PwMS) and which brain areas are the ideal target for stimulation.

Other novel technologies, such as Virtual Reality (VR) and exergames, are emerging as a reinforcing tool to the rehabilitative treatment of PwMS [11,12]. Systematic reviews on VR in rehabilitation across different neurological conditions, such as MS [13], suggest that VR serves as a motivating and engaging method of rehabilitation, thus potentially increasing therapeutic compliance. VR is well-suited to the use of exergames, which are games designed for a purpose beyond simply entertainment. Exergames have demonstrated their utility for both cognitive and motor rehabilitation, also in PwMS [14] as they exert beneficial effects on attention, visuo-spatial function, executive control, strategic planning, and processing speed. Both these novel techniques, tDCS and VR, can be put together in protocols aimed at achieving a better therapeutic benefit across different neurological diseases. Although evidence is still limited, converging findings suggest potential therapeutic benefits of their combined use [15].

In MS, however, literature still lacks strong evidence of efficacy. Therefore, randomized, controlled trials on an adequate number of PwMS are warranted. A preliminary description of the study concept was included in the doctoral dissertation of Lucilla Vestito (University of Genoa, 2023). However, the current manuscript reports the finalized protocol, which was subsequently revised, approved by the Regional Ethics Committee (CET Liguria, No. 447/2023 – DB id 13376), and registered at ClinicalTrials.gov (NCT07114809) [26]. This prompted us to implement a cognitive rehabilitation project in PwMS by combining A-tDCS with exergames. The primary aim is to investigate the potential benefits of combining A-tDCS with exergame-based training in the rehabilitation of CI in PwMS. The secondary aim is to assess whether the proposed protocol can provide sustained benefits over time.

## Materials and methods

### Study design

We planned a double-blind, randomized, prospective, controlled study. A total of 80 MS patients with CI will be recruited.

### Participants

Participants will be recruited from the outpatient services of the Department of Neurology, ASL3 Genoa, the AISM Liguria Rehabilitation Service, and the Neurorehabilitation Unit of IRCCS Ospedale Policlinico San Martino, Genoa. Inclusion criteria are: (1) diagnosis of MS according to McDonald’s criteria [16]; (2) age between 18 and 60 years (to minimize potential age-related CI); and (3) Expanded Disability Status Scale (EDSS) score ≤7.5 [17]. Exclusion criteria include major psychiatric disorders, epilepsy, previous brain surgery, MS relapse requiring steroid therapy within the past two months, and bilateral visual acuity <6/10.

### Outcome measures

Cognitive status will be assessed using the Brief International Cognitive Assessment for MS (BICAMS) and the Paced Auditory Serial Addition Test (PASAT) with 3- and 2-second intervals. The BICAMS battery includes the Symbol Digit Modalities Test (SDMT), the California Verbal Learning Test, 2nd edition (CVLT-II), and the Brief Visuospatial Memory Test-Revised (BVMT-R). All instruments are widely used in cognitive assessment of PwMS. Patients scoring below the 5th percentile of age-, sex-, and education-adjusted norms in at least two of these tests will be eligible. Additional secondary outcomes will include self-reported measures of mood, fatigue, and quality of life: Multiple Sclerosis Quality of Life (MSQoL) [18], Beck Depression Inventory (BDI) [19], and Fatigue Severity Scale (FSS).

### Methodology

Participants will be randomized into two groups: 40 in the experimental group (EG) and 40 in the control group (CG), matched for demographic characteristics (sex, age), EDSS score, and disease duration. All participants will undergo cognitive training using the Neurotablet system, a virtual-reality–based platform incorporating exergames for motor and cognitive rehabilitation. The system includes exercises with adjustable difficulty levels, enabling tailored treatment plans based on individual disability. The rehabilitation program will consist of 10 sessions (1 hour each), delivered 5 days per week for 2 consecutive weeks.

### Intervention treatment

In addition to exergame training, EG participants will receive A-tDCS over the DLPFC, while CG participants will receive sham stimulation (S-tDCS) at the same site. Stimulation will be delivered using a battery-driven constant current stimulator with an LCD touchscreen (HDC progr), a portable stimulator (HDC stim), two cellulose sponge electrodes (7 × 5 cm) in saline bags, and conductive silicone electrodes. The anodal electrode will be placed over the left DLPFC (Brodmann area 46) using a positioning cap, and the reference electrode over the right shoulder. The DLPFC was chosen based on its established role in top-down cognitive control [20], conflict monitoring through anterior cingulate cortex connections [21], and evidence of improved working memory and executive function after A-tDCS in both healthy subjects [22] and PwMS [23]. Conductive gel will be applied under the electrodes to minimize impedance, which will be maintained below 5 kΩ. Only the tDCS operators will know the stimulation condition; participants and neuropsychological assessors will remain blinded. During each exergame session, A-tDCS will be applied online (concurrent with training) at 1.5 mA for 20 minutes, with a current density of 0.06 mA/cm², within established safety limits [4].

### Placebo treatment

In the sham condition, stimulation will be delivered for 30 seconds at the beginning and end of each 20-minute session, producing the typical tingling sensation under the electrodes while preventing sustained cortical modulation. This procedure makes sham indistinguishable from active stimulation, ensuring participant blinding.

### Timeline

**Fig 1:**
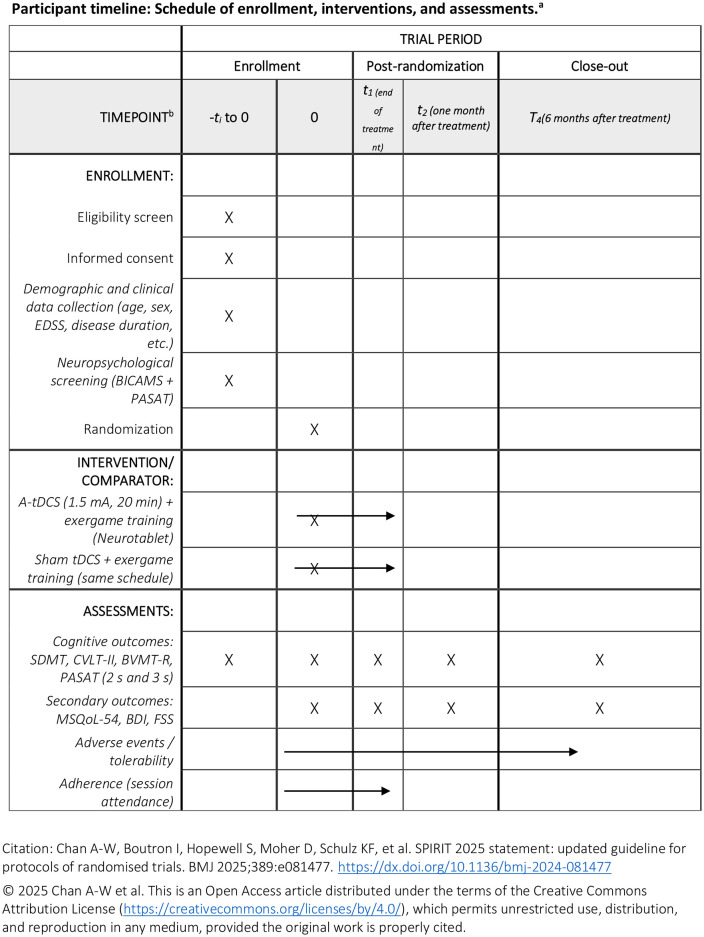
Participant timeline according to the SPIRIT 2025 guidelines.

Outcome measures will be administered at baseline, after the 10 treatment sessions, and at 1-month and 6-month follow-ups. The study duration is estimated at 2 years (Fig 1).

The schedule displays enrollment, randomization, interventions (A-tDCS + exergames; sham tDCS + exergames), and timing of cognitive and secondary outcome assessments at baseline, end of treatment, 1-month, and 6-month follow-ups. Figure adapted from Chan et al., BMJ 2025 (Creative Commons Attribution License).

Patient enrollment began in September 2024 and is expected to be completed by January 2026. Data collection should be finalized by July 2026, and data analysis is anticipated to be completed by September 2026.

### Statistical analysis and sample size

Descriptive characteristics of the included patients will be presented as mean ± standard deviation (SD) or median and interquartile range (IQR) for continuous variables and as absolute frequencies and percentages for categorical variables. Baseline characteristics of the patients will be also shown splitting the patients into the two groups of treatments and comparisons will be performed using Chi-squared test of Fisher’s exact test (for categorical variables) and Mann-Whitney U test or t-test (for continuous variables). Results of the SDMT and PASAT tests will be presented over time as Mean ± SD by showing separately the two groups of treatment. The repeated measures ANOVA will be used to assess the effect of treatment, the effect of time, and the interaction effect between time and treatment on the performance of the SDMT and PASAT tests. The sample size was calculated fixing a power of 0.80 and a significance level of 0.05 and we calculated the sample sizes required for between effect, for within effect and for the between–within interaction effects for both the neuropsychological tests, planning of picking as our final sample size the largest one. To calculate sample sizes, means of measurements over time and covariance matrix for the repeated measures were defined for the SDMT and PASAT tests based on clinical expectations and on previous findings [24]. The largest sample size was the one required for the between effect in the analysis of PASAT test, leading to a sample size of 33 patients in each group to detect the treatment effect of magnitude δ = 0.35. This sample size is even more satisfactory for the SDMT test, where a smaller sample size was required. However, assuming a drop-out rate of 20%, we finally need to enroll in 80 subjects with 40 patients per treatment group. After performing the repeated measures ANOVA, in case of presence of differences, we also plan to compare means using paired t-test, adequately adjusting for multiple testing. In case of missing data over follow-up, a multiple imputation by chained equations approach will be used with 10 imputations. Additionally, we will also perform the same analyses, using complete data to check the robustness of the results. A p value less than 0.05 will be considered significant. All the analyses will be conducted with the Statistical Software Stata version 16.0 (Stata Corporation, College Station, TX, USA). Randomization will be performed using computer-generated random numbers.

### Oversight and monitoring

The IRCCS Ospedale Policlinico San Martino, Genoa, will act as coordinating center, responsible for trial oversight, central randomization, data management, monitoring, and communications with the ethics committee and trial registry. Participants will be recruited at three sites (ASL3 Neurology Department, AISM Liguria Rehabilitation Service, and IRCCS Policlinico San Martino). Each site will have a principal investigator responsible for local conduct. A Steering Committee (PI, site PIs, statistician, data manager) will oversee scientific and operational aspects, including protocol amendments, recruitment, adherence, and data quality. Data will be entered into password-protected eCRFs stored on a secure institutional server, with anonymization, restricted access, and regular back-ups. Given the low-risk nature of the trial, no Data Monitoring Committee is planned; safety will be overseen by the PI and site PIs and reported to the ethics committee.

Participants will be withdrawn in case of intolerable adverse events, clinical worsening requiring treatment changes, participant request, or investigator decision for safety. Stimulation parameters will not be modified during the trial. Adherence will be promoted through flexible scheduling, continuous support, and the engaging use of exergames.

Unblinding is not anticipated but may be authorized by the PI in exceptional safety-related circumstances. In case of discontinuation or deviation, all scheduled outcome data (clinical, neuropsychological, and patient-reported) will continue to be collected and included in intention-to-treat analyses.

All adverse events (AEs), whether solicited or spontaneous, will be documented; minor AEs will be managed immediately, while serious/unexpected AEs will be reported promptly to the PI and ethics committee. Any important protocol modifications will be submitted for ethics approval and updated in the trial registry.

Personal data will be collected only for study purposes, stored securely, and anonymized for analysis/publication. The final dataset will be accessible to the PI and study team only; the sponsor has no restrictions on access. No specific ancillary or post-trial care is planned; clinical needs will be managed as per standard practice. Given the minimal risk profile of tDCS and exergames, no specific compensation beyond national regulations is planned.

Results will be disseminated via peer-reviewed journals, conference presentations, and trial registries. A lay summary of findings will be made available to participants upon request.

### Assessment of capacity to provide consent

All participants will be adults (aged 18–60 years) with a diagnosis of multiple sclerosis but without clinical evidence of severe cognitive or psychiatric impairment preventing autonomous decision-making. Before enrollment, the investigator will ensure each participant’s capacity to provide informed consent through a brief clinical interview and review of recent neuropsychological evaluations. Only individuals who demonstrated adequate understanding of the study’s aims, procedures, and potential risks were included.

The informed consent process, including capacity assessment, was reviewed and approved by the *Comitato Etico Regionale della Liguria* (Ethics Committee approval No. 447/2023 – DB id 13376). As all participants were deemed capable of providing informed consent, no surrogate consent procedures were required.

## Discussion

This project is designed to explore the synergistic effect of A-tDCS and VR-based cognitive rehabilitation in PwMS. By combining two non-conventional restorative approaches, we aim not only to improve cognitive function but also to determine whether the benefits can be sustained over time.

Previous studies have identified key brain regions implicated in attention, information processing and executive function, such as the left DLPFC, cingulate cortices, cerebellum, and parietal areas [1,25]. Our results may contribute to refining this knowledge, clarifying the neural mechanisms underlying CI in MS, and possibly pointing to new therapeutic targets.

Beyond mechanistic insights, integrating A-tDCS with exergames training may promote greater patient engagement, enhancing adherence to cognitive rehabilitation programs. Improved compliance is expected to translate into more durable rehabilitative outcomes and a tangible impact on quality of life.

We anticipate that the combined, simultaneous (on-line) use of A-tDCS and exergames will outperform VR alone (with sham stimulation) in both efficacy and persistence of effects. If confirmed, these findings would strengthen the rationale for implementing integrated neurotechnological protocols in MS rehabilitation, providing protocols that can be translated into everyday rehabilitation settings.

## Supporting information

S1 File Protocol revised. (DOCX)

S2 FIle SPIRIT 2025 participant timeline. (PDF)
