# Supplementary material for: Impact of anodal tDCS and virtual reality on cognitive dysfunction in patients with Multiple Sclerosis: Protocol of a double blind, randomized, prospective, controlled study
Source: PLoS One. 2025 Dec 4;20(12):e0337405. doi: 10.1371/journal.pone.0337405 (PMC12677514; doi:10.1371/journal.pone.0337405)
Supplement: S1 File — (DOCX) [file pone.0337405.s001.docx]

**Title of the study:**

**Impact of anodal tDCS and virtual reality on cognitive dysfunction in patients with Multiple Sclerosis: a double blind, randomized, prospective, controlled study.**

**Acronym:** tDCS Cognitive

#### Type of study

Double blind, randomized, prospective, controlled study

### Duration

Two years

**Centers involved in the study and PI:**

**Unit 1:**

Clinica di Neuroriabilitazione

Ospedale Policlinico San Martino IRCCS

Largo Rosanna Benzi 1, Genova

**Principal Investigator:** Prof. Laura Mori

**Unit 2:**

SC Neurologia ASL3 Genovese

**Principal Investigator:** Dr. Fabio Bandini

**Unit 3:**

Servizio Riabilitazione AISM Liguria

**Principal Investigator:** Dr. Giampaolo Brichetto

**Background, state of the art**

Cognitive impairment (CI) affects up to 70% of the MS population and refers to domain-specific deficits rather than uniform global cognitive decline. Patients with MS (PwMS) may experience difficulties in information processing speed, attention, learning and episodic memory, executive functions and visuospatial abilities. CI can occur even in the early stages of the disease, and approximately half of PwMS report either minimal or mild cognitive difficulties within the first years of diagnosis. CI yields to major consequences for everyday life and is the leading cause of occupational disability and of difficulties in ADLs for PwMS.

The possible therapeutic approaches for CI are mostly rehabilitative. Disease Modifying Therapies might ameliorate CI as they are designed to arrest the course of the disease, but their real benefit on improving cognition is still controversial (McNicholas 2018). The goal of rehabilitative strategies is to reduce cognitive deficits through a specific training and different rehabilitative interventions have been tried in order to alleviate CI, but with limited efficacy (McNicholas 2018). In recent years, transcranial direct current stimulation (tDCS) has emerged as a non-conventional tool in restorative neurology due to its ability in enhancing the efficacy of conventional therapeutic interventions. TDCS treatment consists of applying a direct current flow of low intensity (1-2 mA) over the scalp to modulate cortical excitability, by facilitating or inhibiting ongoing neuronal processes.

The anodal stimulation (A-tDCS) promotes mechanisms that underlie long-term potentiation and triggers widespread cortical activation (Lang 2005). The procedure is well-tolerated, with no evidence of risk for serious adverse effects and no associated risk of seizures (Poreisz 2007). Some side effects as tingling, itching, burning, and pain are seldom reported. These effects are typically mild and fade within 30 to 60 seconds of stimulation. In addition, the potential to include a "placebo" condition (subjects cannot reliably distinguish between real and sham stimulation) makes tDCS the ideal method to reliably investigate its efficacy. Stimulation with tDCS can be implemented using either an *off-line* approach (i.e. stimulating the damaged area at rest, preceded or followed by behavioral intervention), or an *on-line* approach (i.e. stimulating the area of interest while the patient is undergoing a specific rehabilitation training). It is generally assumed that the latter can achieve a better influence on neuroplasticity by targeting specific pools of neurons. Finally, tDCS equipment is relatively inexpensive and easily portable, making it possible for clinicians to administer tDCS to patients in a variety of contexts, including at home. The beneficial effects of tDCS have been reported in a variety of neurological disorders, including MS (Rossini, 2015). In PwMS, tDCS has been applied in alleviating several features of the disease, including pain, gait, spasticity, fatigue and tactile perception (Hiew 2021). Of note, its effects on Cognitive Impairment (CI) have received less attention and, therefore, are not fully elucidated. Mattioli and coworkers (2016) observed a beneficial effect of A-tDCS over the left DLPFC on some cognitive domains.

Grigorescu (2020), on the other hand, found that bifrontal tDCS over the prefrontal cortex (PFC) did not lead to any effect on some cognitive measures. These somehow conflicting studies show that further research is needed to better understand the potential role of tDCS in improving the cognitive performances of PwMS and which brain areas are the ideal target for the stimulation.

Notably, other novel technologies, such as Virtual Reality (VR) and Exergame, are emerging as a reinforcing tool to the rehabilitative treatment of PwMS (Laver 2015, Maggio 2019). VR therapies or interventions are based on real-time motion tracking and computer graphic technologies and can display the patient’s performance during the required task. Systematic reviews on VR in rehabilitation across different neurological conditions, such as MS (Nascimento 2021), suggest that VR serves as a motivating and engaging method of rehabilitation, thus potentially increasing therapeutic compliance. VR is well-suited to the use of exergames, which are games designed for a purpose beyond simply entertainment. The term exergame refers to video games that impart physical exercise/support rehabilitation practice (in the context of their clinical application) in which the repetitive and task-oriented components of rehabilitation activities are reformulated in terms of video game tasks. They are showing promise for teaching new behavior and are being incorporated into more and more rehabilitation therapies with promising results (Cassani 2020). Exergames have demonstrated their utility for both cognitive and motor rehabilitation, also in PwMS (Taylor 2015) as they exert beneficial effects on attention, visuo-spatial function, executive control, strategic planning, and processing speed. The therapies or interventions are based on real-time motion tracking and computer graphic technologies displaying the patient’s performance during the required task in a virtual environment. processing speed.

To take into account the wide variability of patients’ conditions and disease progression, the most appropriate exercises can be assigned and the complexity of the required task can be adapted by defining a range of difficulty levels. Thanks to the collection, integration and remote analysis of patient signals and data, the solution allows continuous monitoring of the activities on which the therapist constantly updates the personalized exercise plan. Based on the clinical needs of PwMS and their specific type of cognitive impairment, new activities delivered in form of exergames have been developed to address functional abilities of attention, memory, executive functions, and information processing speed.

Both these novel techniques, tDCS and VR, can be put together in protocols aimed at achieving a better therapeutic benefit across different neurological diseases. There is indeed converging evidence of the therapeutics benefits of their combined use (Cassani 2020). In MS, however, the literature still lacks strong evidence of efficacy, and only a single case report has been published so far (Costa 2019). As a consequence, randomized, controlled trials on an adequate number of PwMS are warranted. This prompted us to implement a cognitive rehabilitation project in PwMS by combining A-tDCS with the exergames training in a group of PwMS.

To our knowledge, this study will be the first attempt to treat CI in a large group of PwMS by means of the

concurrent use of A-tDCS and VR.

**Rationale and specific aims**

Cognitive impairment (CI) is one of the multiple symptoms that can affect a MS patient, compromising daily living activities (ADL) and social interactions (McNicholas 2017). As already stated, many papers investigate this aspect of the disease, unexpectedly identifying a large proportion of affected individuals (40-82%). The possible therapeutic approaches are mostly neuropsychological rehabilitation treatment, since pharmacological and immunosuppressive therapies did not show significant results (McNicholas 2017). Rehabilitation aims to increase the patient’s awareness of their own CI and create mechanisms for them to manage with CI in daily life, but especially to reduce cognitive deficits through cognitive retraining. Before undergoing the rehabilitation treatment, it is very important to accurately test the cognitive sphere to clearly identify if the impairment is in attention, working memory, information processing speed, visuospatial or executive deficits (Nocentini 2006).

Non-invasive brain stimulation (NIBS) techniques have recently emerged in restorative neurology due to their hypothetical advantage in enhancing the efficacy of traditional therapeutic intervention, since they are

recognized as safe and relatively painless instrument for manipulating performance in a variety of motor and cognitive domains. They are generally represented by repetitive transcranial magnetic stimulation (rTMS) and transcranial direct current stimulation (tDCS). In MS, the use of NIBS as a rehabilitation tool is raising much interest. Treatment with rTMS (Palm 2014; Meesen 2014) has been successfully applied in motor dexterity, spasticity, urinary symptoms and fatigue, while tDCS has been reported to exert beneficial effects on pain (Ayache 2017), fatigue Lefaucheur 2017, Ayache 2017), tactile perception (Mori 2013), cognition, attention and executive function (Mattioli 2016). The tDCS treatment consists of applying a direct current flow of low intensity (1-2 mA) over the scalp to modulate cortical excitability by facilitating or inhibiting ongoing neuronal processes. It acts by a tonic modulation of the resting membrane potential of the cortical neurons. Cathodal stimulation (CtDCS) decreases cortical excitability due to neural hyperpolarization, while anodal stimulation (A-tDCS) reaches the opposite effect by a subtreshold depolarization (Nitche 2000). Particularly A-tDCS has been shown to induce beneficial effects on multiple aspects of the disease and, moreover, on cognitive aspects. Particularly, Mattioli and his co-workers, in 2016, submitted a group of MS patients to an intensive cognitive training program to improve attention and information processing speed and an active treatment of A-tDCS or S-tDCS over the left DLPFC; A-tDCS increased the efficacy of cognitive training used to improve attention and information processing deficits immediately after the treatment lasting six months after. Moreover, patients in the active stimulation group reached the most difficult training level faster than those in the sham group. VR has several advantages compared to classical cognitive rehabilitation tasks in designing effective cognitive training. VR has an enhanced ecological validity and offers the possibility to provide immediate performance feedback, which is generally accepted to be necessary for most forms of learning and for successful rehabilitation. Moreover, VR offers the possibility to personalize the environment and the activities, to make them more engaging thus enhancing the compliance to the treatment. To the best of our knowledge, no study investigated the combined effect of a cognitive treatment performed with VR and A-tDCS (Cassani 2020).

Aim of our project is to explore the potential benefits of the simultaneous application of A-tDCS and VR in the rehabilitation of cognitive impairment of PwMS. The VR approach will be implemented through a novel exergames training.

As a secondary outcome, we wish to verify whether our protocol may extend its benefits over a long period (6 months) For this purpose, we will recruit 80 PwMS affected by CI from two centers specialized in the management of multiple sclerosis: Department of Neurology of the ASL3 of Genoa and Neurorehabilitation unit, IRCCS Ospedale Policlinico San Martino of Genoa.

We suggest that the use of exergames system, with a suite of real-life tasks to train working memory, visuospatial orientation, selective attention tasks, recognition memory tasks, and calculation, can help subjects to better comprehend the assigned exercises thus obtaining increased compliance to the treatment. This could be measured thanks to the chosen outcome scales, evaluating the impact of the cognitive rehabilitation on the quality of life and subjective mood.

**Preliminary results**

Our group has been studying the effect of tDCS on patients affected by different neurological disorders since the pioneering work of Vestito in 2014. In that study three patients affected by chronic post-stroke aphasia underwent anodal tDCS (A-tDCS, 20 min, 1.5 mA) and sham stimulation (S-tDCS) over the left frontal (perilesional) region, coupled with a simultaneous naming training (on-line tDCS). Ten consecutive sessions (five days per week for two weeks) were implemented. In the first five sessions we used a list of 40 figures, while in the subsequent five sessions we utilized a second set of 40 figures differing in word difficulty. At the end of the stimulation period we found a significant beneficial effect of A-tDCS (as compared to baseline and S-tDCS) in all our subjects, regardless of word difficulty. In the follow-up period, the percentage of correct responses persisted significantly better until the 16th week, when an initial decline in naming performance was observed. That study showed that repetitive sessions of A-tDCS over the Broca’s area, at a variance with placebo, produced a significant and long-lasting (16 weeks) improvement in naming performances of aphasic patients. We then confirmed the positive impact of A-tDCS in a single case study of post-stroke thalamic aphasia. In this case, the peculiarity of our study relied upon the rehabilitative intervention in a subacute phase of the disease, while it is generally assumed that brain stimulation should be applied in chronically impaired patients. In addition, a cortical electrical stimulation such as tDCS interestingly extended its beneficial effect to a subcortical structure (the thalamus) or to the thalamo-cortical projections (Campanella 2020).

We then extended our research interest on the rehabilitative yield of tDCS in some neurodegenerative diseases, such as Posterior Cortical Aphasia (PCA). We indeed demonstrated, for the first time, that the combination of a computer-assisted cognitive training, together with a simultaneous A-tDCS over the Parietal Posterior Cortex (PPC), induced a significant amelioration of visuo-spatial neglect (VN), compared to baseline and cognitive treatment alone (Vestito 2021).

The same approach was followed in another study on two post-stroke patients with VN. This study confirmed the positive results of our rehabilitative strategy, which combines the exergames with a concurrent A-tDCS (Trombini 2020). These promising beneficial effects prompted us to verify the efficacy of this approach on the cognitive deficits of PwMS. In a pilot single-blind, sham-controlled study, we enrolled 6 PwMS patients, partitioned in two groups. The first group (3 subjects) underwent multiple sessions (10) of A-tDCS (20 min, 1,5 mA) over the left DLPFC coupled with the computer assisted training (CAT). The second group underwent the same protocol coupled with a sham stimulation (S-tDCS). The cognitive disorder was assessed one week before the experimental session using a neuropsychological battery (BICAMS, PASAT 2”-3”). The same evaluation was repeated at the end of the stimulation period. The CAT and the simultaneous A-tDCS and S-tDCS were administered for 20 minutes for two weeks (5 days for week). The efficacy of the treatment was determined by an improvement of the neuropsychological tests of at least 30% from the baseline values. At the end of the stimulation period, we found a beneficial effect of BICAMS measures (but not on PASAT) for patients treated with combined A-tDCS and CAT as compared to baseline and to patients who underwent S-tDCS and CAT.

**Inclusion criteria:**

- Confirmed diagnosis of MS
- Adults ranging from 18 and 60 yo
- EDSS ≤ 7.5
- Score below the 5th percentile on the following tests:
  - BICAMS
  - PASAT

**Exclusion Criteria:**

- Severe mood disorder;
- Steroid therapy in the 2 months preceding the visit
- history of epilepsy;
- Presence of pacemakers, defibrillators and cochlear implants.
- Presence of intracranial metal plates
- Visual acuity < 6/10

**Research plan and methods**

To achieve our aims, we planned a double-blind, randomized, prospective, controlled study. To this purpose, we will recruit 80 MS subjects affected by cognitive impairment (CI). Patients will be selected from outpatients attending the Department of Neurology of ASL3 Genoa and the Neurorehabilitation unit of IRCCS Ospedale Policlinico San Martino, Genoa.

The inclusion criteria will be the following: 1) MS diagnosis according to McDonald’s criteria (McDonald 2017); 2) age between 18 and 60 (to avoid participants with possible CI due to aging); 3) disability score ≤7.5 at the Expanded Disability Status Scale (EDSS, Kurtzke 1983). We will exclude subjects affected by major psychiatric disorders, epilepsy, previous brain surgery, MS relapse requiring steroid therapy in the previous two months, bilateral visual acuity < 6/10.

**Outcome measures:**

The Brief International Cognitive Assessment for MS (BICAMS) and the Paced Auditory Serial Addition Task 3” and 2” intervals (PASAT) will be employed as neuropsychological battery. The BICAMS test includes the Symbol digit modalities test (SDMT), the California Verbal Learning test II edition (CVLT-II) and the Revised Brief Visuo-Spatial Memory test (BVMT-R). Both tests are widely utilized for the cognitive assessment in PwMS. Patients will be recruited if they reach scores below the 5th percentile for normative data adjusted for age, sex and education in at least two of the above-mentioned tests. A written, informed consent will be obtained from participants prior to the beginning of the study. Self-reported measures of mood (depression), fatigue and QoL will be included as other secondary outcomes: Multiple Sclerosis Quality of life (MSQoL - Solari 1999); the Beck depression inventory scale (BDI – Beck 1961); the Fatigue Severity Scale (FSS).

All the above-mentioned cognitive measures and the self-reported scales will be administered at baseline, at the end of the 10 treatment sessions, one month and six months after the end of treatment.

**Methodology:**

The participants will be randomly assigned to two groups, 40 in the experimental group (EG), 40 in the control group (CG), matched for demographic data (gender, age), EDSS and disease duration. All subjects will undergo a cognitive training by means of the exergames system (10 sessions, one hour per session, 5 days per week, for two consecutive weeks). Patients in the EG group will undergo a simultaneous A-tDCS over the left DLFPC, while CG will receive a S-tDCS over the same area. The tDCS will be delivered by a battery-driven, constant current simulator with a LCD touch screen (HDC progr), a portable stimulator (HDC stim), two holding bags of plant cellulose (7x5 cm) and two electrodes of conductive silicone. The active (anodal) electrode will be placed by means of a cap on the scalp overlying the left DLPFC (46 Brodmann Area). The reference electrode will be located over the right shoulder. The choice of the left DLPFC as the site of stimulation relies upon the evidence that this region has a critical role in the “top-down” control of the task-relevant stimuli processing (Miller 2001). In addition, in tasks where a cognitive conflict arises, the DLPFC contributes to increased cognitive control through its connections with the anterior cingulate cortex (Botvinick 2001). Finally A-tDCS of the DLPFC has been shown to enhance working memory and executive function in healthy subjects (Fregni 2005) as well as in PwMS (Mainero 2004). An electroconductive gel will be applied under the electrodes in order to reduce contact impedance. Impedance will be constantly kept below 5 kOhm.

Only the tDCS investigators will be aware of the type of stimulation, while the patients and the neuropsychological assessors will be blind as to the nature of the project.

During the exergames training (on-line procedure), A-tDCS (current of 1,5 mA) will be delivered for 20 minutes, while maintaining the current density (0.06 mA/cm2) below the safety limits (Poreisz 2007).

**Placebo treatment:**

In the StDCS session (20 minutes) the current will be turned off 30 sec after the beginning of the stimulation and turned on for the last 30 sec. By doing this, the patient feels an itching sensation below the electrodes at the beginning and at the end of stimulation, making this condition indistinguishable from the real (anodic) stimulation. Doing this, all the subjects will be blinded on the type of stimulation (anodal or sham).

**Cognitive training:**

All the patients will participate to the cognitive training by means of exergames, which includes motor and cognitive tasks that incorporate enjoyment, technology, and health care. The platform will process all the data to make them clear and available within the Therapist Client, processed as a set of graphs and values. This type of layout provides analysis from a cognitive and rehabilitation perspective and allows the multidimensional team to interpret the information more easily. All data and signals are collected and accessible to clinical staff through the Therapist to acquire feedback on activity performance.

Exergames are digital games that encourage patient to carry out motor/cognitive exercises.

The new cognitive games introduced here aim to treat some of the most common symptoms of multiple sclerosis such as coordination disorders and dizziness; vision disturbances which may also include impaired color vision, cognitive disorders that incorporate problems with memory and learning, difficulties in maintaining concentration, difficulties in attention, in computational problems; and inability to perform operations of a certain complexity and in problems to correctly perceive the environment. In all the exercises there are different levels, from easy to extremely difficult. This is in order to define the treatment plan based on the patients’ disability, aimed at selecting the most appropriate game and level to start and continue therapy.

We expect to collect clinical, patient-oriented and instrumental data to show that the combined approach of two novel, non-traditional restorative methodologies, namely A-tDCS and VR, will show a significant and long-lasting beneficial effect on cognitive function in PwMS. In particular, we expect that a simultaneous (on-line) approach of A-tDCS with VR training will lead to better rehabilitative results as compared to the VR rehabilitation alone (i.e. with S-tDCS), in terms of efficacy and duration. A significant amelioration in PwMS QoL is also expected.

**Statistical analysis and sample size**

Descriptive characteristics of the included patients will be presented as mean ± standard deviation (SD) or

median and interquartile range (IQR) for continuous variables and as absolute frequencies and percentages for categorical variables. Baseline characteristics of the patients will be also shown splitting the patients into the two groups of treatments and comparisons will be performed using Chi-squared test of Fisher’s exact test (for categorical variables) and Mann-Whitney U test or t-test (for continuous variables). Results of the SDMT and PASAT tests will be presented over time as Mean ± SD by showing separately the two groups of treatment. The repeated-measures ANOVA will be used to assess the effect of treatment, the effect of time, and the interaction effect between time and treatment on the performance of the SDMT and PASAT tests. The sample size was calculated fixing a power of 0.80 and a significance level of 0.05 and we calculated the sample sizes required for between effect, for within effect and for the between–within interaction effects for both the neuropsychological tests, planning of picking as our final sample size the largest one. To calculate sample sizes, means of measurements over time and covariance matrix for the repeated measures were defined for the SDMT and PASAT tests based on clinical expectations and on previous findings (Naeeni Davarani 2020). The largest sample size was the one required for the between effect in the analysis of PASAT test, leading to a sample size of 33 patients in each group to detect the treatment effect of magnitude δ=0.35. This sample size is even more satisfactory for the SDMT test, where a smaller sample size was required. However, assuming a drop-out rate of 20%, we finally need to enroll 80 subjects with 40 patients per treatment group. After performing the repeated measures ANOVA, in case of presence of differences, we also plan to compare means using paired t-test, adequately adjusting for multiple testing. In case of missing data over follow-up, a multiple imputation by chained equations approach will be used with 10 imputations. Additionally, we will also perform the same analyses, using complete data, to check the robustness of the results. A p value less than 0.05 will be considered significant. All the analyses will be conducted with the Statistical Software Stata version 16.0 (Stata Corporation, College Station, TX, USA).

**Expected results:**

we hypothesize that the cognitive performances of both EG and CG groups will show an improvement in the cognitive performances. We will expect, however, a significative difference between the two groups, with patients in the EG group demonstrating better results than the CG group. Finally, we hypothesize the beneficial effects in EG patients will last at least one month after the end of the experiment.

**Relevance for multiple sclerosis**

Cognitive impairment, particularly deficits in attention, working memory, information processing speed and executive functions, affects a large amount of PwMS (40%-70%, Benedict 2020) even in the early stages of disease. Recent studies have investigated the positive effect of computer support or exergame associated with the cognitive rehabilitation (Amato 2014; De Giglio 2015, Dardiotis 2018, Manuli 2020). Mattioli in 2016 combined a cognitive treatment with A-tDCS over the dorso-lateral prefrontal cortex in 20 MS patients, finding significant improvement in attention and executive functions with durable effects. In our project we aim to confirm their results and verify if the combined use of tDCS and VR solutions, may increase the efficacy of the cognitive treatment, could ameliorate the adherence and the comprehension of the treatment, so enhancing the possible improvements.

**Proximity to find new pathogenic mechanism**

Literature tells us that areas involved in attention tasks, information processing and executive function are left DLPFC, cingulate cortices and cerebellum (Filippi 2012), while other authors report the involvement of the posterior cerebral lobule and superior parietal lobule (McNicholas 2017). The results of the present project may confirm these results allowing for a better comprehension of the dysfunction pattern. Understanding the dysfunction underlying mechanisms can direct research towards new potential therapy targets, allowing more targeted rehabilitation programs.

**Proximity to cure**

This project aims to perform a cognitive rehabilitation treatment specific for cognitive dysfunction, demonstrated to be present even in the early stages of disease. These deficits affect ADLs, limiting personal independence in many activities such as working, driving or having social interactions. A-tDCS is a safe and effective treatment, already used for motor symptoms (Meesen 2014), fatigue (Lefaucheur 2017, Ayache 2017), and pain (Ayache 2016) in PwMS and in other neurological diseases. Recent works proved A-tDCS to have durable effects on PwMS cognitive affected subjects (Charvet 2017, Mattioli 2016). We suggest that A-tDCS associated with VR cognitive rehabilitation by mean of exergames training could improve the adherence to the cognitive treatment, leading to more durable effects and significant repercussion on PwMS quality of life.

**Bibliography**

1. Cassani R, Novak GS, Falk TH, Oliveira AA. Virtual reality and non-invasive brain stimulation for rehabilitation applications: a systematic review. J Neuroeng Rehabil. **2020**, 31;17(1):147
2. Taylor, M.J.D.; Griffin, M. The use of gaming technology for rehabilitation in people with multiple sclerosis. *Mult. Scler.* **2015**, *21*, 355–371, doi:10.1177/1352458514563593.
3. Lang, N.; Siebner, H.R.; Ward, N.S.; Lee, L.; Nitsche, M.A.; Paulus, W.; Rothwell, J.C.; Lemon, R.N.;

Frackowiak, R.S. How does transcranial DC stimulation of the primary motor cortex alter regional neuronal

activity in the human brain? *Eur. J. Neurosci.* **2005**, *22*, 495–504, doi:10.1111/J.1460-9568.2005.04233.X.

1. Manuli, A.; Maggio, M.G.; Tripoli, D.; Gullì, M.; Cannavò, A.; La Rosa, G.; Sciarrone, F.; Avena, G.; Calabrò, R.S. Patients’ perspective and usability of innovation technology in a new rehabilitation pathway: An exploratory

study in patients with multiple sclerosis. *Mult. Scler. Relat. Disord.* **2020**, *44*,

doi:10.1016/J.MSARD.2020.102312.

1. Dardiotis, E.; Nousia, A.; Siokas, V.; Tsouris, Z.; Andravizou, A.; Mentis, A.F.A.; Florou, D.; Messinis, L.; Nasios, G. Efficacy of computer-based cognitive training in neuropsychological performance of patients with multiple sclerosis: A systematic review and meta-analysis. *Mult. Scler. Relat. Disord.* **2018**, *20*, 58–66,

doi:10.1016/J.MSARD.2017.12.017.

1. De Giglio, L.; De Luca, F.; Prosperini, L.; Borriello, G.; Bianchi, V.; Pantano, P.; Pozzilli, C. A Low-Cost Cognitive Rehabilitation With a Commercial Video Game Improves Sustained Attention and Executive Functions in Multiple Sclerosis. *Neurorehabil. Neural Repair* **2015**, *29*, 453–461, doi:10.1177/1545968314554623.
2. Gholami, F.; Trojan, D.A.; Kovecses, J.; Haddad, W.M.; Gholami, B. A Microsoft Kinect-Based Point-of-Care Gait Assessment Framework for Multiple Sclerosis Patients. *IEEE J. Biomed. Heal. Informatics* **2017**, *21*, 1376–1385, doi:10.1109/JBHI.2016.2593692.
3. Trombini, M.; Ferraro, F.; Iaconi, G.; Vestito, L.; Bandini, F.; Mori, L.; Trompetto, C.; Dellepiane, S. A Study Protocol for Occupational Rehabilitation in Multiple Sclerosis. *Sensors (Basel).* **2021**, *21*, doi:10.3390/S21248436.
4. Miller, E.K.; Cohen, J.D. An Integrative Theory of Prefrontal Cortex Function. *Annu. Rev. Neurosci.* **2001**, *24*, 167–202, doi:10.1146/annurev.neuro.24.1.167.
5. Fregni, F.; Boggio, P.S.; Nitsche, M.; Bermpohl, F.; Antal, A.; Feredoes, E.; Marcolin, M.A.; Rigonatti, S.P.; Silva, M.T.A.; Paulus, W.; et al. Anodal transcranial direct current stimulation of prefrontal cortex enhances working memory. *Exp. Brain Res.* **2005**, *166*, 23–30, doi:10.1007/s00221-005-2334-6.
6. McNicholas, N.; O’Connell, K.; Yap, S.M.; Killeen, R.P.; Hutchinson, M.; McGuigan, C. Cognitive dysfunction in early multiple sclerosis: a review. *QJM An Int. J. Med.* **2018**, *111*, 359–364, doi:10.1093/qjmed/hcx070.
7. Botvinick, M.M.; Braver, T.S.; Barch, D.M.; Carter, C.S.; Cohen, J.D. Conflict monitoring and cognitive control.
8. *Psychol. Rev.* **2001**, *108*, 624–52.
9. Morando, M.; Ponte, S.; Ferrara, E.; Dellepiane, S. Definition of Motion and Biophysical Indicators for Home-
10. Based Rehabilitation through Serious Games. *Inf. 2018, Vol. 9, Page 105* **2018**, *9*, 105,
11. doi:10.3390/INFO9050105.
12. Seeley, W.W.; Menon, V.; Schatzberg, A.F.; Keller, J.; Glover, G.H.; Kenna, H.; Reiss, A.L.; Greicius, M.D.
13. Dissociable intrinsic connectivity networks for salience processing and executive control. *J. Neurosci.* **2007**, *27*,
14. 2349–56, doi:10.1523/JNEUROSCI.5587-06.2007.
15. Nascimento, A.S.; Fagundes, C.V.; Mendes, F.A. dos S.; Leal, J.C. Effectiveness of Virtual Reality Rehabilitation
16. in Persons with Multiple Sclerosis: A Systematic Review and Meta-analysis of Randomized Controlled Trials.
17. *Mult. Scler. Relat. Disord.* **2021**, *54*, doi:10.1016/J.MSARD.2021.103128.
18. Grigorescu, C.; Chalah, M.A.; Lefaucheur, J.P.; Kümpfel, T.; Padberg, F.; Ayache, S.S.; Palm, U. Effects of
19. Transcranial Direct Current Stimulation on Information Processing Speed, Working Memory, Attention, and
20. Social Cognition in Multiple Sclerosis. *Front. Neurol.* **2020**, *11*, doi:10.3389/FNEUR.2020.545377.
21. Hiew, S.; Nguemeni, C.; Zeller, D. Efficacy of transcranial direct current stimulation in people with multiple sclerosis: a review. *Eur. J. Neurol.* **2022**, *29*, 648–664, doi:10.1111/ENE.15163.
22. Nitsche, M.A.; Paulus, W. Excitability changes induced in the human motor cortex by weak transcranial direct
23. current stimulation. *J. Physiol.* **2000**, *527*, 633–639, doi:10.1111/j.1469-7793.2000.t01-1-00633.x.
24. Mainero, C.; Caramia, F.; Pozzilli, C.; Pisani, A.; Pestalozza, I.; Borriello, G.; Bozzao, L.; Pantano, P. fMRI
25. evidence of brain reorganization during attention and memory tasks in multiple sclerosis. *Neuroimage* **2004**, *21*,
26. 858–867, doi:10.1016/j.neuroimage.2003.10.004.
27. Vestito, L.; Trombini, M.; Mori, L.; Dellepiane, S.; Trompetto, C.; Morando, M.; Bandini, F. Improved visuospatial neglect after tDCS and computer-assisted cognitive training in Posterior Cortical Atrophy: a single-case study. *Neurocase* **2021**, *27*, 57–63, doi:10.1080/13554794.2020.1862242.
28. Vestito, L.; Rosellini, S.; Mantero, M.; Bandini, F. Long-Term Effects of Transcranial Direct-Current Stimulation in Chronic Post-Stroke Aphasia: A Pilot Study. *Front. Hum. Neurosci.* **2014**, *8*, doi:10.3389/fnhum.2014.00785.
29. Filippi, M.; Riccitelli, G.; Mattioli, F.; Capra, R.; Stampatori, C.; Pagani, E.; Valsasina, P.; Copetti, M.; Falini, A.; Comi, G.; et al. Multiple sclerosis: effects of cognitive rehabilitation on structural and functional MR imaging measures--an explorative study. *Radiology* **2012**, *262*, 932–40, doi:10.1148/radiol.11111299.
30. Mattioli, F.; Bellomi, F.; Stampatori, C.; Capra, R.; Miniussi, C. Neuroenhancement through cognitive training and anodal tDCS in multiple sclerosis. *Mult. Scler.* **2016**, *22*, 222–30, doi:10.1177/1352458515587597.
31. Naeeni Davarani, M.; Arian Darestani, A.; Hassani-Abharian, P.; Vaseghi, S.; Zarrindast, M.R.; Nasehi, M.
32. RehaCom rehabilitation training improves a wide-range of cognitive functions in multiple sclerosis patients. *Appl.*
33. *Neuropsychol. Adult* **2022**, *29*, doi:10.1080/23279095.2020.1747070.
34. Charvet, L.; Shaw, M.; Dobbs, B.; Frontario, A.; Sherman, K.; Bikson, M.; Datta, A.; Krupp, L.; Zeinapour, E.;
35. Kasschau, M. Remotely Supervised Transcranial Direct Current Stimulation Increases the Benefit of At-Home
36. Cognitive Training in Multiple Sclerosis. *Neuromodulation Technol. Neural Interface* **2018**, *21*, 383–389,
37. doi:10.1111/ner.12583.
38. Poreisz, C.; Boros, K.; Antal, A.; Paulus, W. Safety aspects of transcranial direct current stimulation concerning
39. healthy subjects and patients. *Brain Res. Bull.* **2007**, *72*, 208–14, doi:10.1016/j.brainresbull.2007.01.004.
40. Wood, J.; Finkelstein, J. Telerehabilitation system to support multipronged exercise in patients with multiple
41. sclerosis. *Proc. - 2017 IEEE Int. Conf. Bioinforma. Biomed. BIBM 2017* **2017**, *2017*-*January*, 880–885,
42. doi:10.1109/BIBM.2017.8217772.
43. Trombini, M.; Vestito, L.; Morando, M.; Mori, L.; Trompetto, C.; Bandini, F.; Dellepiane, S. Unilateral spatial
44. neglect rehabilitation supported by a digital solution: two case-studies. *Annu. Int. Conf. IEEE Eng. Med. Biol.*
45. *Soc. IEEE Eng. Med. Biol. Soc. Annu. Int. Conf.* **2020**, *2020*, 3670–3675,
46. doi:10.1109/EMBC44109.2020.9175834.
47. Solari, A.; Filippini, G.; Mendozzi, L.; Ghezzi, A.; Cifani, S.; Barbieri, E.; Baldini, S.; Salmaggi, A.; Mantia, L.L.;
48. Farinotti, M.; et al. Validation of Italian multiple sclerosis quality of life 54 questionnaire. *J. Neurol. Neurosurg.*
49. *Psychiatry* **1999**, *67*, 158–162, doi:10.1136/jnnp.67.2.158.
50. Cassani, R.; Novak, G.S.; Falk, T.H.; Oliveira, A.A. Virtual reality and non-invasive brain stimulation for
51. rehabilitation applications: a systematic review. *J. Neuroeng. Rehabil.* **2020**, *17*, doi:10.1186/S12984-020-
52. 00780-5.
53. Laver, K.E.; George, S.; Thomas, S.; Deutsch, J.E.; Crotty, M. Virtual reality for stroke rehabilitation. *Cochrane*
54. *Database Syst. Rev.* **2015**, doi:10.1002/14651858.CD008349.pub3.
