# Supplementary material for: Impact of anodal tDCS and virtual reality on cognitive dysfunction in patients with Multiple Sclerosis: Protocol of a double blind, randomized, prospective, controlled study
Source: PLoS One. 2025 Dec 4;20(12):e0337405. doi: 10.1371/journal.pone.0337405 (PMC12677514; doi:10.1371/journal.pone.0337405)
Supplement: S2 FIle — (PDF) [file pone.0337405.s002.pdf]

Participant timeline: Schedule of enrollment, interventions, and assessments.[a]

	TRIAL PERIOD				
	Enrollment		Post-randomization		Close-out
TIMEPOINT[b]	$-t_i$ to 0	0	t_1 *(end of treatment)*	t_2 *(one month after treatment)*	T_4*(6 months after treatment)*
ENROLLMENT:					
Eligibility screen	X				
Informed consent	X				
Demographic and clinical data collection (age, sex, EDSS, disease duration, etc.)	X				
Neuropsychological screening (BICAMS + PASAT)	X				
Randomization		X			
INTERVENTION/ COMPARATOR:					
A-tDCS (1.5 mA, 20 min) + exergame training (Neurotablet)		X →	→		
Sham tDCS + exergame training (same schedule)		X →	→		
ASSESSMENTS:					
Cognitive outcomes: SDMT, CVLT-II, BVMT-R, PASAT (2 s and 3 s)	X	X	X	X	X
Secondary outcomes: MSQoL-54, BDI, FSS		X	X	X	X
Adverse events / tolerability		→	→	→	→
Adherence (session attendance)		→	→		

Citation: Chan A-W, Boutron I, Hopewell S, Moher D, Schulz KF, et al. SPIRIT 2025 statement: updated guideline for protocols of randomised trials. BMJ 2025;389:e081477. https://dx.doi.org/10.1136/bmj-2024-081477

© 2025 Chan A-W et al. This is an Open Access article distributed under the terms of the Creative Commons Attribution License (https://creativecommons.org/licenses/by/4.0/), which permits unrestricted use, distribution, and reproduction in any medium, provided the original work is properly cited.
